# Evidence levels in radiology: the insights into imaging approach

**DOI:** 10.1186/s13244-021-00995-7

**Published:** 2021-04-07

**Authors:** Luis Martí-Bonmatí

**Affiliations:** Hospital Universitario Y Politécnico La Fe, Valencia, Spain

Medicine is based on knowledge from scientific studies and the validation of clinical experience. Medical knowledge must be well established before it can be considered as the basis for decision making. Journals have a clear responsibility to help readers recognize the level of evidence for the claims published in their manuscripts. Levels of evidence alone do not determine the quality of the article but help readers to understand the significance of the claims.

We recognize that our discipline, radiology and medical imaging, also suffers from a certain lack of reproducibility of its results when translated into practice. Although our clinical work is firmly based on years of practice and well-known criteria and characteristics, new proposals and some older standards are not free of errors and biases.

This is the main reason why our journal encourages authors to follow this guideline when analyzing referenced papers and their own work (Table 1). The level and confidence in the evidence and the degree of consideration of the recommendations and their wordings are based on the type and quality of the references and the results of the paper. *Authors are encouraged to specify in the manuscript the appropriate level and recommendation of their claims, following the criteria of this journal*. The categorization into only three levels is based on publications in addition to critical approach to technical and clinical studies related to medical imaging [1–5]. These levels attempt to reconcile scientific knowledge and clinical certainty. We hope that this classification and grading will enlighten readers to better understand the relevance of published results and claims.

**Table 1:** Levels of evidence and recommendation

Levels of evidence and recommendation		
Level of evidence		Confidence in the evidence and recommendations grade
High	Data derived from metanalyses or systematic reviews or from (multiple) randomized trials with high quality Large retrospective observational studies or in silico clinical trials with external validation Well defined reference standards and controlled biases The described technique improves healthcare pathway (tests, treatment, hospitalization) or decreases costs per patient Level is graded down to Moderate if there are limiting biases or inconsistencies between studies	Further research is unlikely to change our confidence in the estimate of benefit and risk Strongly recommended, mainly if presumed important patient outcomes and/or acceptable costs Wording associated with the High grade of recommendation: ‘‘must”, ‘‘should” “recommend”
Moderate	Data derived from a single large randomized clinical trial or multiple nonrandomized studies Large retrospective observational multicentre studies or large in silico clinical trials with controlled design and internal validation. Appropriate spectrum of cases Studies on technique assessments of noninferiority, surrogate biomarkers or changes in clinical management Level can be upgraded to High if there is a demonstrated large effect size or downgraded if the effect size is small	Further research is likely to have an impact on our confidence in the estimate of benefit and risk and may change the estimate Recommendation is modulated to strong or weak by the presumed patient outcomes and final costs
Low	Small series, non-validated results and single centre observational, experimental or technical studies None or imperfect reference standards No study on the validation of results Large possible biases Opinions, general statements, critical and educational reviews without analytical methods Studies on either technical efficacy or diagnostic validation accuracy (reference standards)	Any estimate of effect is uncertain Weak recommendation, mainly if not clear patient important outcomes and/or high cost Wording associated with the Low grade of recommendation: ‘‘could”, ‘‘may”, “suggests”

## Data Availability

All relevant data are published in this Editorial.
